# A Plea for Early Surgical Interference in Pelvic Infections

**Published:** 1907-02

**Authors:** 


### A PLEA FOR EARLY SURGICAL INTERFERENCE IN PELVIC INFECTIONS.

J. C. Taylor believes that the best results are attained in pelvic infections by attacking inflammatory conditions early. Conservative operations may sometimes fail; if they do, radical procedures may be resorted to later without any added risk to the patient. In regard to gonorrheal infection the writer believes that pain and muscular rigidity are sufficient evidence that the tubes are already involved. He describes the operation of opening and draining the tubes. In those cases in which the inflammation has been under palliative treatment for a long time, and in which, upon late examination a large pus sac is revealed, conservatism cannot be adopted with any degree of success. In cases of staphylococcic and streptoccocic infection after abortion or labor the diagnosis and surgical interference must be made early or the post mortem examination will show the mistake.—*Medical Record, November* 17, 1906.

